# Probe Sector Matching for Freehand 3D Ultrasound Reconstruction

**DOI:** 10.3390/s20113146

**Published:** 2020-06-02

**Authors:** Xin Chen, Houjin Chen, Yahui Peng, Dan Tao

**Affiliations:** School of Electronic and Information Engineering, Beijing Jiaotong University, Beijing 100044, China; hjchen@bjtu.edu.cn (H.C.); yhpeng@bjtu.edu.cn (Y.P.); dtao@bjtu.edu.cn (D.T.)

**Keywords:** 3D ultrasound imaging, 3D ultrasound reconstruction, freehand acquisition, 3D Hough transform, coordinate transformation

## Abstract

A 3D ultrasound image reconstruction technique, named probe sector matching (PSM), is proposed in this paper for a freehand linear array ultrasound probe equipped with multiple sensors, providing the position and attitude of the transducer and the pressure between the transducer and the target surface. The proposed PSM method includes three main steps. First, the imaging target and the working range of the probe are set to be the center and the radius of the imaging field of view, respectively. To reconstruct a 3D volume, the positions of all necessary probe sectors are pre-calculated inversely to form a sector database. Second, 2D cross-section probe sectors with the corresponding optical positioning, attitude and pressure information are collected when the ultrasound probe is moving around the imaging target. Last, an improved 3D Hough transform is used to match the plane of the current probe sector to the existing sector images in the sector database. After all pre-calculated probe sectors are acquired and matched into the 3D space defined by the sector database, a 3D ultrasound reconstruction is completed. The PSM is validated through two experiments: a virtual simulation using a numerical model and a lab experiment using a real physical model. The experimental results show that the PSM effectively reduces the errors caused by changes in the target position due to the uneven surface pressure or the inhomogeneity of the transmission media. We conclude that the PSM proposed in this study may help to design a lightweight, inexpensive and flexible ultrasound device with accurate 3D imaging capacity.

## 1. Introduction

Ultrasound imaging plays an important role in clinical diagnosis, in which the locations of the abnormalities need to be detected accurately [1,2]. Compared with other diagnostic methods, ultrasound imaging has several advantages, including its promptness, non-invasiveness and low cost. In addition, the ultrasound imaging probe works with low power consumption and causes no harm to patients and operators. Therefore, ultrasound-based diagnostic methods are used widely for screening and preventive health care, such as the regular prenatal care checkups for pregnant women and their fetuses.

A drawback of traditional 2D ultrasound imaging is that only cross-sectional images of the anatomical target are provided. Doctors need to reconstruct the full 3D structure of the target in mind. Additionally, it can be difficult to place the 2D transducer in a perfect position to appreciate the ideal cross section [3]. 3D reconstruction [4,5,6,7] may help solve this problem by synthesizing a set of cross sections with the spatial position information recorded by the transducer during the imaging. Reconstructed 3D images help doctors to better understand the anatomical morphology more intuitively. However, current equipment in the market with 3D ultrasound imaging capacity is expensive and often has a limited field of view.

Therefore, freehand 3D ultrasound reconstruction has become a research topic in recent years [8,9,10,11,12,13,14,15,16,17,18,19,20,21,22,23,24,25,26,27,28,29,30,31,32,33], aiming to address the concerns of cost and field of view more appropriately. The freehand technology reduces the cost by using a linear transducer instead of a multi-dimensional transducer array in most 3D ultrasound products. Additionally, the linear transducer design is more compact, so the manipulation of the transducer becomes more convenient, and the field of view can be improved as well.

In general, there are three main ways to implement 3D ultrasound reconstruction: 1) A freehand system equipped with multiple positioning sensors [8,9,11,16,17,18,19,20,21,26]. This design is relatively lightweight. However, when the transducer is moved, the measured target surface may also be squeezed, generating distortions. Consequently, the position of the target may be displaced, as well, and the medium between the target and the probe changes. These two problems will eventually lead to the loss of fidelity. 2) A 3D robotic arm is used instead of a free hand to drive the transducer to translate, tilt and rotate [12,14,15,22,23,24,33]. However, it is difficult to improve the spatial resolution in this way, and the equipment is bulky and difficult to operate. 3) Multiple linear array transducers may be used simultaneously to acquire a set of cross sections distributed in different locations in space [31,32]. In this way, the equipment is often large and costly.

Based on the three designs mentioned above, a number of studies were carried out. An adaptive kernel regression method was proposed for volume reconstruction from freehand 2D images. The purpose of the method was to prevent the reconstructed image from being degenerated by speckle noise and artifacts [8]. By comparing the results of the reconstruction algorithm with the ultrasound data source, differences in image quality of the reconstructed volumes could be detected. This method generated new approaches for improving the quality of 3D reconstruction [11]. At low acquisition frame rates, a probe trajectory-based reconstruction method was able to improve the reconstruction results, but it was limited to the case where the probe moved at a constant speed [15]. In order to overcome the effects of target surface changes, a section-locating method based on Hough transform was proposed, which was able to quickly locate the current transducer sector by comparing the points obtained from the Hough transform with the actual position of the transducer [16]. By controlling the contacting force between the ultrasound probe and the surface of the target, a handheld force-controlled ultrasound probe was developed to improve the stability of ultrasound images [17]. Because this probe combined force and position control, it might help to overcome the effects caused by the surface changes. However, when the probe was in contact with a rigid target surface, the self-jitter of the probe would cause errors. In order to improve the reconstruction accuracy of the ultrasound image, the freehand probe was installed on a shelf with position sensors and multiple motion motors. The shelf was used to limit the movement of the probe. This method improved the quality of the reconstructed 3D images, but the portability and flexibility of the device limited the applications of this design [22].

In light of the advantages and disadvantages of the three common methods mentioned above, the current study proposes a freehand 3D ultrasound image reconstruction method, named probe sector matching (PSM), based on the linear array transducer design. PSM includes three main steps: 1) With the target as the imaging center, and the working range of the probe as the radius, all the 2D image planes that the probe sector may pass are obtained first to enable the fast matching algorithm implemented in a later stage [34,35,36,37,38]. 2) The user holds the probe and moves freely on the surface of the measured target. During the movement, the probe produces a set of 2D ultrasound images. An optical positioning device and an attitude sensor installed on the probe are used to obtain the high-precision spatial position and attitude of the probe. At the same time, a pressure sensor measures the pressure between of the probe and the target surface. This pressure is used to compensate the position errors caused by the squeezing. 3) With the improved 3D Hough transform [39,40,41], the current probe sector plane corresponds to one of the planes pre-calculated in Step 1. All the probe sectors are collected and aligned in a 3D space to complete the 3D ultrasound reconstruction. PSM is tested in two experiments, one with a numerical model and the other with a real target.

## 2. Space Plane Inverse Operation

**Figure 1:**
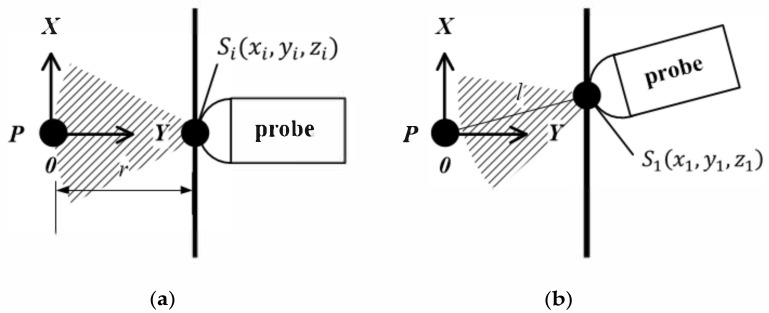
The probe and the imaging target in ideal situation: (**a**) Distance is equal to the working radius of the ultrasound probe; (**b**) Distance is not equal to the working radius of the ultrasound probe.

In the proposed freehand 3D ultrasound imaging system with a linear-transducer probe, in which a space positioning device is installed, reconstruction of the 3D image based on the obtained 2D images and the actual position of the probe sector is the most critical technique. The working radius of the ultrasonic probe is generally fixed for the depth parameter of the same target. During the freehand movement of the probe, the distance between the imaging target and the probe changes. In case the surface of the target is rigid, the distance between the probe and the measured part may still vary due to changes in the position and angle of the probe itself, as shown in Figure 1.

In Figure 1, the shaded sector is the effective detection range of the probe, P is the location of the imaging target, r is the working radius of the probe, and $S_{i}\left(x_{i},y_{i},z_{i}\right)$ are the probe coordinates obtained by the external positioning sensor. The probe moves along the surface of the target. When the probe moves to a new position, $S_{1}\left(x_{1},y_{1},z_{1}\right)$, the distance l may not be equal to the working radius of the ultrasound probe anymore.

**Figure 2:**
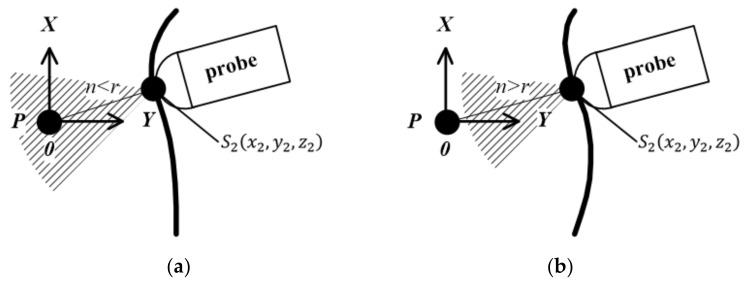
Surface of the target changes: (**a**) Distance less than the radius; (**b**) Distance is greater than the radius.

In practice, the probe squeezes the surface of the target to be imaged during the movement. The distance between the probe and the target may change, causing the target to move in or out of the working radius of the probe at a certain time. However, due to the uncertainty of distance changes, direct calculations have a lot of extra overhead, as shown in Figure 2.

In actual measurement, the target position $P_{i}\left(x,y,z\right)$ needs to be set first. Assuming that the target is a straight line L, in a Cartesian coordinate system, we define L by the following equations:

(1) $$\left\{\begin{array}{c} A_{1}x+B_{1}y+C_{1}z+D_{1}=0\\ A_{2}x+B_{2}y+C_{2}z+D_{2}=0 \end{array}\right.$$

$A_{1},B_{1},C_{1}\;\mathrm{and}\;D_{1}$ are parameters for Plane 1 and $A_{2},B_{2},C_{2}\;\mathrm{and}\;D_{2}$ are parameters for Plane 2. It is assumed that the ultrasonic waves enter in a direction perpendicular to the Z axis. Then, the sector obtained by the linear transducer may exist in countless planes passing through the straight line L. For the sake of convenience, assume that the line L overlaps with the Z axis. To locate the line *L*, the intersection of the sector and the XY plane is needed. The method of calculating the spherical coordinates of the existing radius can be obtained by leveraging the idea of line detection based on Hough transform in a 2D plane.

**Figure 3:**
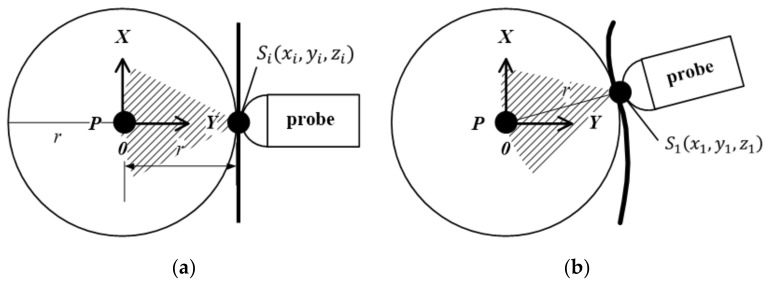
Method based on Hough transform: (**a**) Target surface unchanged; (**b**) Target surface change.

As shown in Figure 3, in a spherical coordinate system with P as the origin of the coordinates, $S_{i}\left(x_{i},y_{i},z_{i}\right)$ is a point on the sphere with a distance r from the origin P of the coordinates. If the detection depth of the probe is also set to be r, then $S_{i}\left(x_{i},y_{i},z_{i}\right)$ are the coordinates of the contact point between the probe and the target surface. According to Hough transform, in the case where the radius r is known and P is the origin of the spherical coordinate system, a set of points $\left\{S_{1}\left(x_{1},y_{1},z_{1}\right),\ldots ,S_{n}\left(x_{n},y_{n},z_{n}\right)\right\}$ can be calculated.

**Figure 4:**
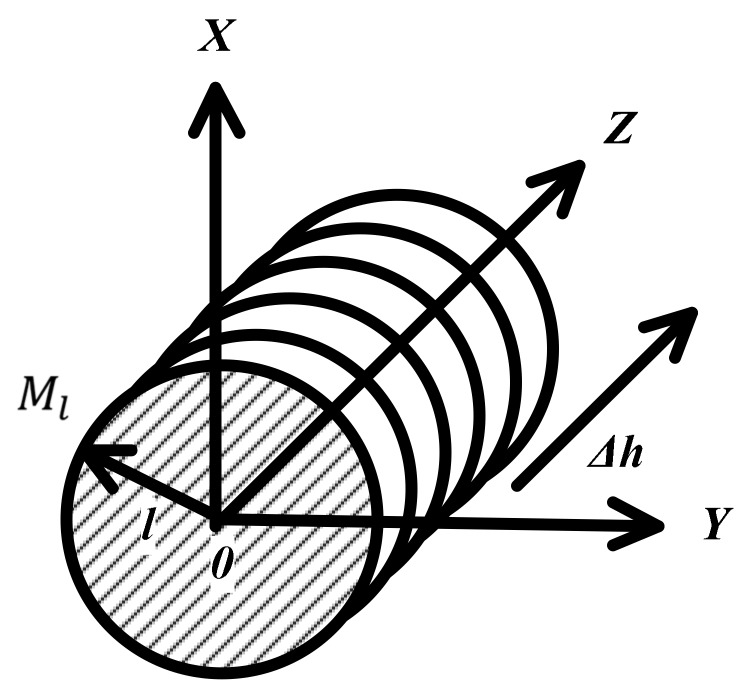
Horizontal resolution of measured target.

Expanded into a 3D space, with P as the origin of Cartesian coordinates, *XYZ* as the axis direction and the target $M_{l}$ as a cylinder with a radius l, the straight line L mentioned earlier passes through the center of the cylinder, as shown in Figure 4. Δh is the horizontal resolution of the linear transducer. Δh is generally a constant, related to the number of line transducers. It may also be changed by moving the probe along the Z axis.

**Figure 5:**
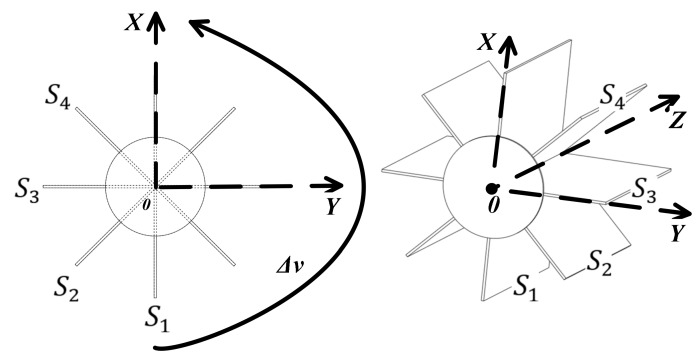
Vertical resolution of the measured target.

Supposing that the surface of the measured target is a cylinder and that the straight line L passes through the center axis of the cylinder, with Equation (1), four straight lines can be calculated on the surface of the cylinder that are parallel to L. In Figure 5, the four planes composed of these four straight lines and the straight line L divide the cylinder into eight equal parts.

With any two points coming from the four sectors that pass L:$\;S_{1},S_{2},S_{3}\;\mathrm{and}\;S_{4}$—for example, points $A\left(x_{1},y_{1},z_{1}\right)$ and $B\left(x_{2},y_{2},z_{2}\right)$ on L, and another point $P_{i}\left(x_{i},y_{i},z_{i}\right)$ in each sector—the equation of each sector obtained by the coordinates of three points is as follows:

(2) $$\left|\begin{array}{ccc} x-x_{1} & y-y_{1} & z-z_{1}\\ x_{2}-x_{1} & y_{2}-y_{1} & z_{2}-z_{1}\\ x_{i}-x_{1} & y_{i}-y_{1} & z_{i}-z_{1} \end{array}\right|=0$$

where $x_{i},y_{i}\;\mathrm{and}\;z_{i}$ are the coordinates of any point $P_{i}\left(x_{i},y_{i},z_{i}\right)$ in the sector, $P_{1}\left(x_{1},y_{1},z_{1}\right)$ is one point in $S_{1}$, $P_{2}\left(x_{2},y_{2},z_{2}\right)$ is one point in $S_{2}$, and so on.

The number of sectors in Figure 5 is equal to the vertical resolution Δv. The more sectors, the better the quality of the reconstructed image but the longer it takes for the space plane inverse operation, which is important for creating the sector locations, and the more storage space it needs to consume.

The steps of the space plane inverse operation are shown in Table 1.

## 3. Spatial Position and Attitude of Probe Sector

### 3.1. Optical Positioning of the Probe

In order to get the spatial position of the freehand probe, PSM uses the optical positioning method with virtual reality equipment. This positioning system includes an external base station and multiple photosensors. The base station has an infrared LED array and two infrared laser emitters that rotate perpendicularly to each other. In order to obtain accurate position information, ultrasound or other positioning technologies are sometimes used to assist.

When the base station is operating, two infrared laser emitters alternately emit light during a rotating cycle. Multiple optical sensors are mounted on an optical positioning ball, which is attached to the freehand probe. Once three or more photosensitive sensors receive signals from the base station at the same time, we can measure the time when the two infrared lasers reach the sensor. Combined with the rotation angle information, the spatial location of the positioning ball on the freehand probe can be calculated, and subsequently, the trajectory of the probe can be obtained as a time sequence.

**Figure 6:**
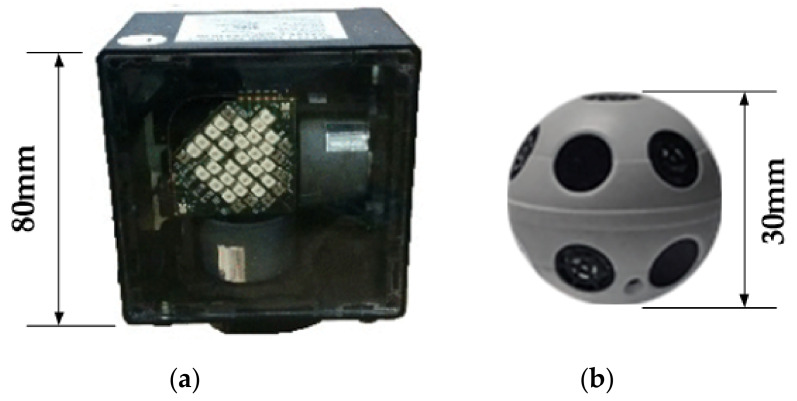
Optical positioning of the probe: (**a**) Base station; (**b**) The optical positioning ball.

The accuracy of this positioning method depends on the temporal resolution. There is a certain distance between the light-sensitive sensors to ensure that the information of the rotation angle does not appear too large. This positioning method has many advantages: 1) the device is small in size; 2) the computational complexity is low; 3) the delay is small; and 4) the position feedback is accurate. It has been widely used in virtual reality equipment. The optical positioning ball and base station used in this paper are shown in Figure 6.

**Figure 7:**
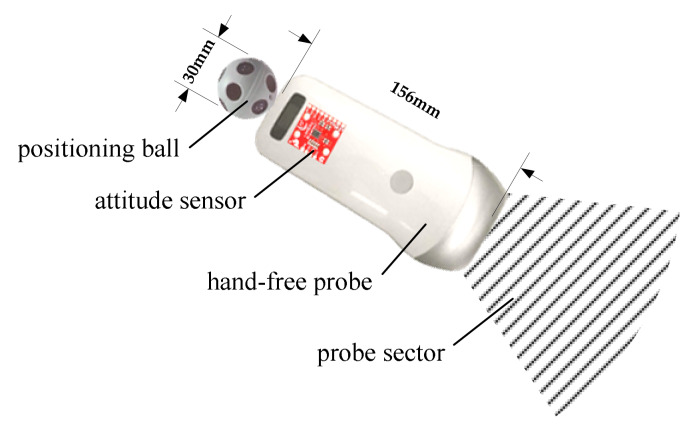
Probe with the attitude sensor and the positioning ball.

Although the positioning ball and the base station can be used to obtain the spatial position and moving trajectory of the probe, the spatial attitude of the probe cannot be determined yet. To that end, an attitude sensor is also required. The installation position of the attitude sensor and the positioning ball on the probe is shown in Figure 7. The 2D ultrasound images collected by the current sector of the probe will be transmitted wirelessly to a computer for processing.

### 3.2. Pressure Sensor of the Probe

**Figure 8:**
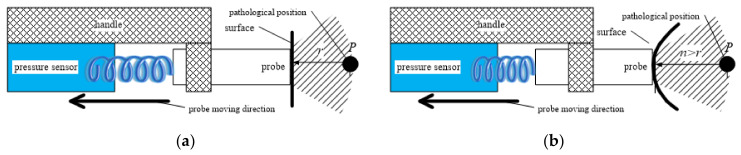
The probe with a pressure sensor. (**a**) Surface is not squeezed; (**b**) Surface is squeezed.

The initial working radius of the ultrasound probe is r. As the freehand probe moves along the surface of the measured target, the ultrasonic transmission medium may change. The pressure of the probe on the surface may cause the target to change its position. Because the change of the medium cannot be predicted, the working radius of the probe cannot be adjusted, either. This situation sometimes leads to the situation in which the acquired ultrasound image does not include the target, making the 3D synthesis difficult. Adding a handle with a pressure sensor to the probe effectively solves the positioning problem caused by the change of the medium. Figure 8 is a schematic diagram of the pressure sensor installation. The greater the density of the medium, the greater the force feedback when the probe is squeezed on the surface. The medium from the linear transducer to the target location is usually non-linear. The feedback pressure roughly estimates the average medium density of the current detection area, which is crucial for post-processing.

In Figure 8, the target position changes when the probe is held against the surface with the handle. At this time, the working radius of the probe needs to be increased to acquire a 2D ultrasound image including the target for 3D reconstruction. The feedback value of the pressure sensor guides the adjustment of the working radius of the probe.

### 3.3. Error Analysis of the Sector

**Figure 9:**
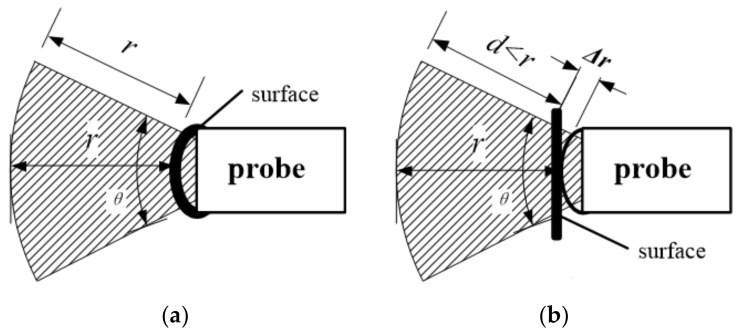
Error analysis of the sector: (**a**) the probe fits the surface; (**b**) the probe does not fit the surface.

Ideally, the initial working radius of the ultrasound probe is set to r. In the actual operation, the working radius of the ultrasound image sector actually collected by the probe is not necessarily equal to r. The working radius mainly depends on whether the linear transducer completely fits the surface of the measured target. Because the force of the squeeze is not controllable, the working radius of the sector edge is not necessarily equal to r. There is a certain error Δr, as shown in Figure 9.

Figure 9a shows that the probe is close to the surface of the object to be measured, and the working radius of the sector is r. Figure 9b shows that the probe is not close to the surface. The working radius of the center of the sector is r, but the working radius of the edge becomes d=r−Δr. In this case, the error of different line transducers is different. The sector error can be expressed as $\Delta r_{1},\Delta r_{2},\Delta r_{3},\ldots ,\Delta r_{n}$, where n is the total number of probe linear transducers. The error value can be calculated from the arrangement curve of the transducer. Squeezing the probe to get the pressure and comparing it with the error value may reduce the error.

## 4. Improved 3D Hough Transform

The Hough transform can transform the global detection problem in the image space into a local peak detection problem in the parameter space. The Hough transform identifies and detects any analytical curve in the image space. The essence of the Hough transform is the mapping from image space to parameter space, that is, all points on the analytical curve in image space are concentrated to a certain unit in the parameter space to form a local peak. As long as there are enough data points in the image space that belong to the same analytical curve, the parameters of the analytical curve can be calculated by judging the accumulated value of each point in the parameter space.

To extend the 2D Hough transform to 3D, we need to determine an appropriate straight-line transform method in a 3D space. Because the parameter range of Cartesian coordinates is not bounded, it is necessary to convert the straight-line representation of a 2D straight line from the Cartesian coordinates to a spherical coordinate system. The conversion Equation is as follows.

(3) xsinθcosφ+ysinθsinφ+zcosθ=r

where r is the distance from the origin to the target point, θ is the angle between the straight line and the positive direction of the Z-axis, and φ is the projection of the line on the XY plane and the positive angle of the X-axis. $\left\{P_{1}\left(x_{1},y_{1},z_{1}\right),\ldots ,P_{n}\left(x_{n},y_{n},z_{n}\right)\right\}$ is the set of points corresponding to XYZ space, which satisfies the following restrictions.

First, let us take two points, $A\left(a_{1},a_{2},a_{3}\right)$ and $B\left(b_{1},b_{2},b_{3}\right)$ from the probe sector. Substitute the coordinates of each point of the $\left\{P_{1}\left(x_{1},y_{1},z_{1}\right),\ldots ,P_{n}\left(x_{n},y_{n},z_{n}\right)\right\}$ set into Equation (3), according to the restrictions in Table 2. By substituting $A\left(a_{1},a_{2},a_{3}\right)$ and $B\left(b_{1},b_{2},b_{3}\right)$ into Equation (3), two more equations can be obtained. Now, we have three equations to calculate the value of $\left(\theta ,\;\varphi ,\;r\right),$ as shown in Equation (4). After all points are used, the coordinates of a certain position in the space are obtained, and the accumulator of this position is incremented by one. Each point is traversed once to complete the 3D Hough transform of all the points. Taking the position with the largest count, the corresponding $\left(\theta ,\;\varphi ,\;r\right)$ can be converted to the plane data in XYZ space.

(4) $$\left\{\begin{array}{c} a_{1}\sin\theta \cos\varphi +a_{2}\sin\theta \sin\varphi +a_{3}\cos\theta =r\\ b_{1}\sin\theta \cos\varphi +b_{2}\sin\theta \sin\varphi +b_{3}\cos\theta =r\\ x_{n}\sin\theta \cos\varphi +y_{n}\sin\theta \sin\varphi +z_{n}\cos\theta =r \end{array}\right.$$

**Figure 10:**
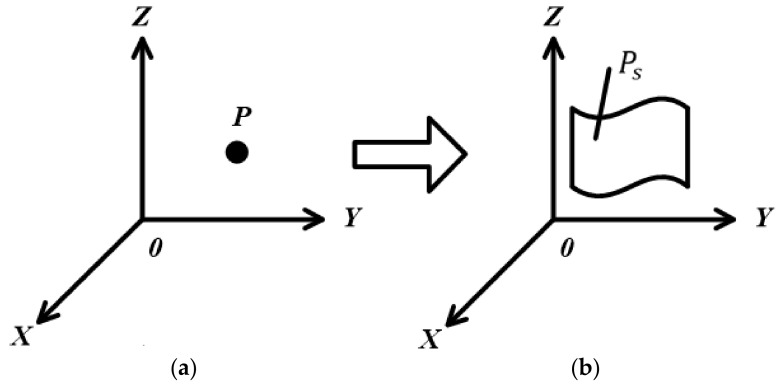
The 3D Hough transform: (**a**) The parameter space; (**b**) The image space.

In the 3D Hough transform, the point P of the maximum value of the straight line in the parameter space corresponds to a plane $P_{s}$ of the coordinates, as shown in Figure 10. In Figure 10a, P is the maximum accumulated value in the parameter space. After 3D Hough transform, one can get a plane in the image space, as shown in Figure 10b.

Different representations of coordinate space correspond to different 3D Hough transforms. It needs to be selected comprehensively from the aspects of calculation complexity and storage capacity according to the actual situation. It can be seen from Equation (4) that the unknown variables of the expressions in coordinate space and parameter space are the same; all of them are three. Therefore, when we want to reduce the calculation dimension, we can consider reducing the number of unknown variables in coordinate space. The fewer the unknown variables of the expressions in coordinate space, the fewer the calculation dimensions of the corresponding parameter space. Based on the principle of the inverse Hough transform, this paper proposes an improved 3D Hough transform method. Points A, B and C in the coordinate space correspond to the planes $A_{s}$, $B_{s}$ and $C_{s}$ in the parameter coordinates.

**Figure 11:**
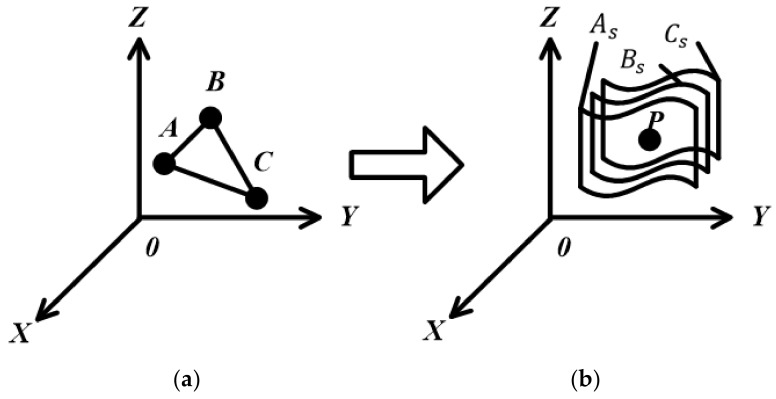
The improved 3D Hough transform: (**a**) The parameter space; (**b**) The image space.

As shown in Figure 11b, P is the intersection of $A_{s}$, $B_{s}$ and $C_{s}$ in image space. According to the 3D Hough transform, we can get a plane composed of three points A, B and C in the parameter space, as shown in Figure 11a. In other words, the intersection point P of planes $A_{s}$, $B_{s}$ and $C_{s}$ is the description of the plane where points A, B and C are located. In this way, the plane data of any three points in space coordinates can be obtained.

**Figure 12:**
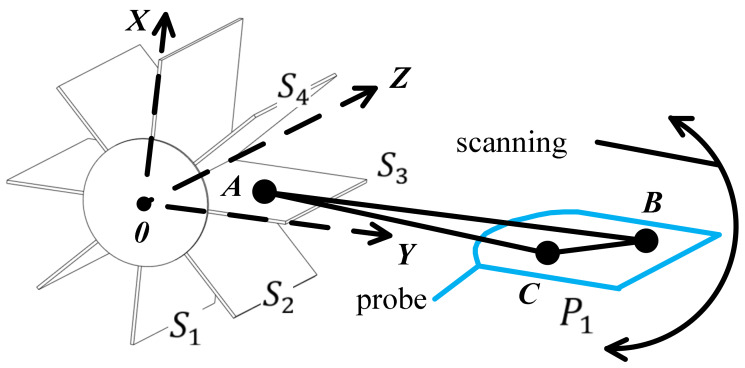
Probe sector synthesis in a 3D space.

In Figure 12, the plane data, $S_{1},\;S_{2},\;S_{3}\;and\;\;S_{4}$ that the probe may pass through are obtained by inverse calculation from the target position. The freehand probe moves in the space, and the space plane $P_{1}$ where the current sector is located is obtained by the positioning device. The core of 3D reconstruction is to quickly and accurately place 2D images of multiple probe sectors into a 3D space. Through the improved 3D Hough transform in this paper, the current sector picture of the probe can be quickly placed into the spatial plane model.

Steps of the improved 3D Hough transform proposed in this paper are shown in Table 3.

## 5. Reconstruction Experiment

The Probe Sector Matching (PSM) proposed in this paper is verified by two methods: a numerical model and a real target. First, a numerical model with adjustable surface and medium parameters is established with MATLAB, using the k-wave toolbox to create multiple probes that simulate the movement of a freehand probe in a 3D space. Pressure feedback and position feedback are obtained through the PSM, which is used to modify the distorted reconstructed image. Then, the correct reconstructed image is obtained. For the second method, the real target is placed in a hemispherical container filled with a sound coupling medium. A wireless linear probe with a spatial positioning device is used to acquire ultrasound images. The positioning device is installed on the wireless probe, which is composed of the attitude sensor and positioning ball. The process of obtaining the probe position is described in the third part. A set of images including the spatial positions collected by the probe are transmitted to the computer by wireless transmission. These images obtained are reconstructed through PSM to reconstruct the real target with MATLAB.

### 5.1. Experiment Preparation

**Figure 13:**
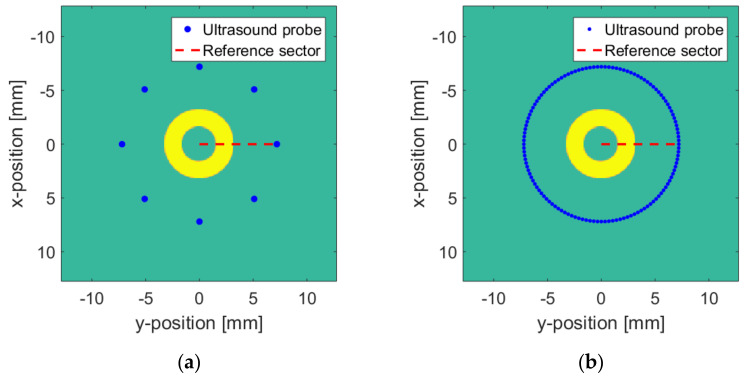
Numerical model: (**a**) Δv  = 8; (**b**) Δv = 128.

The numerical model is based on the Huygens principle, which means that when a sound wave encounters an obstacle, the obstacle will become a new sound source. First, a circular target is created in the *XY* plane. The spatial positions of the target and the probe are shown in Figure 12. According to Figure 5, two sets of ultrasonic sensors with vertical resolutions of 8 and 128 are established to simulate the freehand movement of the probe in the 3D space. The vertical resolution is the number of ultrasonic sensors, and it is also the location where the ultrasonic probe collects images in space. As shown in Figure 13a, if the vertical resolution = 8, then the ultrasonic probe will collect images at eight positions with average distribution in the space. As shown in Figure 13, the blue dots are the positions passed by the probe. The surface where the probe moves is the initial position of the blue dot. The red dotted line is the reference sector, which is used for data normalization when the surface and medium change. The center of the yellow ring target is located at the origin of the XY plane.

**Figure 14:**
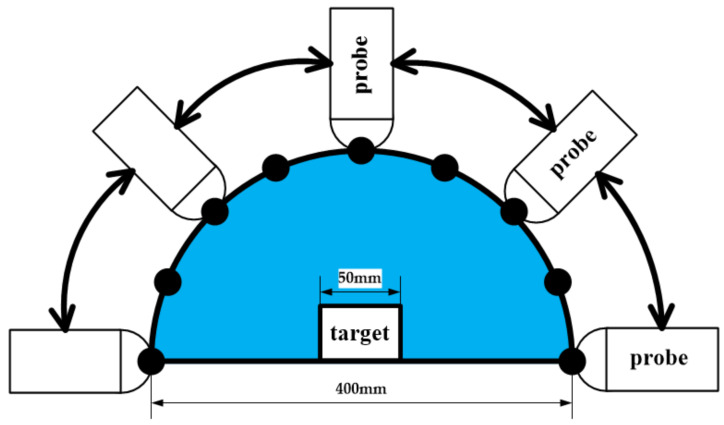
The experiment with the real model.

Table 4 describes the specification of the wireless linear ultrasound probe used in the real model experiment. The real model is also a ring-shaped object, which is placed in a hemispherical container filled with the coupling medium, as shown in Figure 14. The probe moves along the surface of the container to acquire a set of ultrasound images, which is used as the raw data for the PSM.

### 5.2. Numerical Model Reconstruction Result

**Figure 15:**
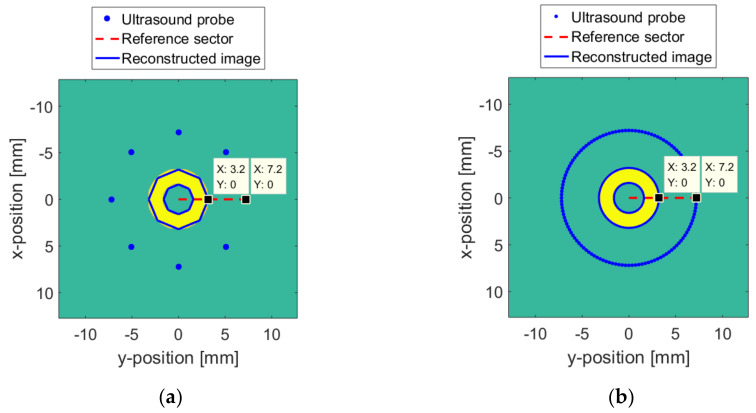
The numerical model: (**a**) Δv  = 8; (**b**) Δv = 128.

**Figure 16:**
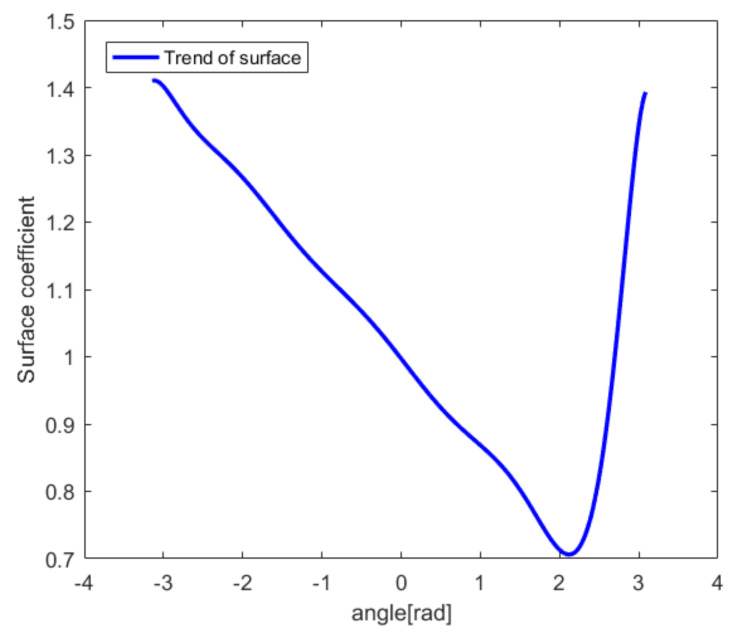
Surface change curve.

In the ideal case where the surface and the medium are unchanged, the relative position between the yellow ring target and the probe is unchanged. The detection radius r of the probe is set to a constant greater than n. Acquisition and processing are completed at vertical resolutions of 8 and 128, respectively. Then, the image of the yellow ring target is synthesized, which is indicated by the blue line in Figure 15. The higher the vertical resolution, the closer the target image is to reality. It can be seen from the data cursor on the reference sector in Figure 16 that the distance between the probe and the ring target is n = 4 mm.

Based on the initial values in Table 5, interpolation and fitting methods are used to establish a continuously changing surface parameter curve, as shown in Figure 16. The horizontal axis represents the change in radians. The vertical axis represents the surface coefficient of variation.

Table 5 shows the initial values of the numerical model when the moving surface of the probe changes. The resolution Δv is 8. The speed of sound is 1500 m/s. The frequency is 1 kHz. n is the distance between the probe and the target on the reference sector, which is 4 mm. On the reference sector, it takes 2.7 μs for the probe to receive the echo. Therefore, 2.7 μs is the reference time. When the surface changes, the echo time error is relative to 2.7 μs. In order to simulate surface changes, the initial value of D is between 1.75 mm and 7 mm, which also indicates the change in the distance between the probe and the target. T and Δt represent the time and error of the probe receiving the echo.

**Figure 17:**
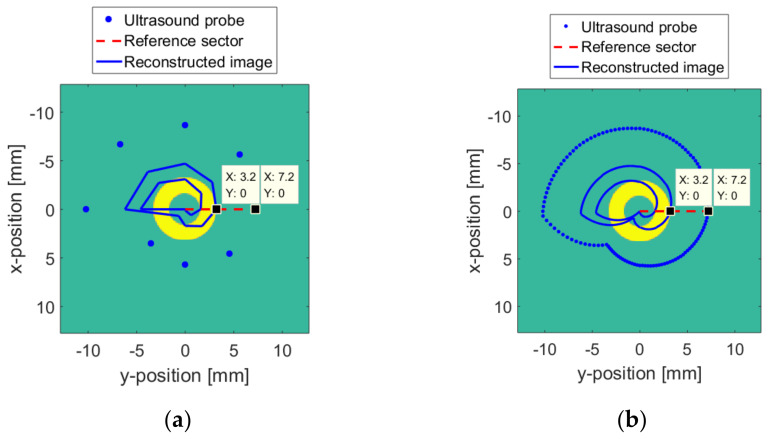
Reconstructed image when the surface changes without the probe sector matching (PSM): (**a**) Δv = 8; (**b**) Δv = 128.

When the surface changes according to Figure 16, the ideal reconstruction algorithm cannot compensate the error by adjusting the working parameters of the probe. Therefore, except for the reference sector, the reconstructed images of other probe positions are not aligned correctly. The final reconstructed image is shown by the blue line in Figure 17.

The relative position of the probe and the target is D = 4 mm and remains unchanged. However, the transmission medium coefficient (TMC) of the probe changes. The initial values when the TMC changes in the numerical model are shown in Table 6. On the reference sector, the value of TMC is initialized to 1 and is used as the reference value of the echo time error when the TMC changes. The TMC values range from 2.77 to 0.75. The larger the value of the TMC, the weaker the transmission capacity of the medium and the longer the time for the echo to travel through the medium.

**Figure 18:**
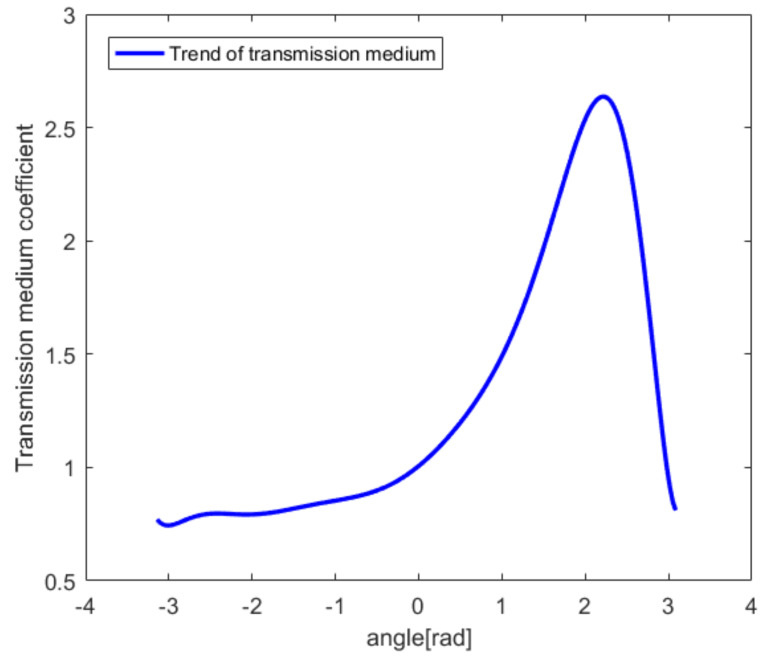
The transmission medium coefficient (TMC) curve.

**Figure 19:**
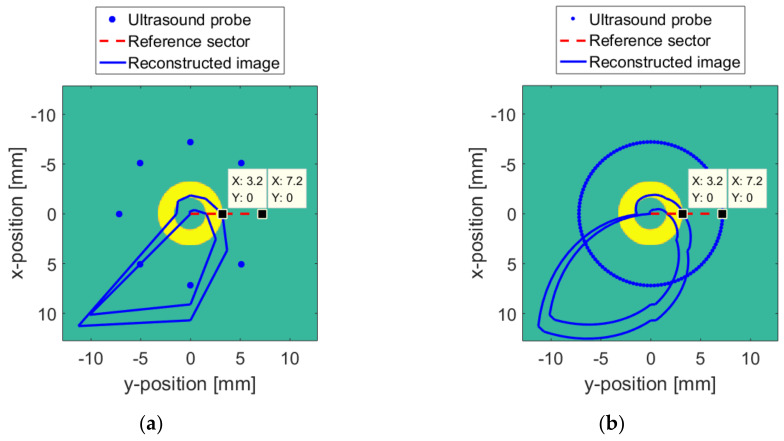
Reconstructed image when the TMC changes without the PSM: (**a**) Δv = 8; (**b**) Δv = 128.

Similarly, on the basis of Table 6, interpolation and fitting are used to establish a continuously changing TMC parameter curve, as shown in Figure 18. The horizontal axis represents the change in radians. The vertical axis represents the transmission medium coefficient. When the TMC changes according to Figure 18, the final reconstructed image without the PSM is shown by the blue line in Figure 19, in which (a) and (b) are the results for Δv = 8 and Δv = 128.

**Figure 20:**
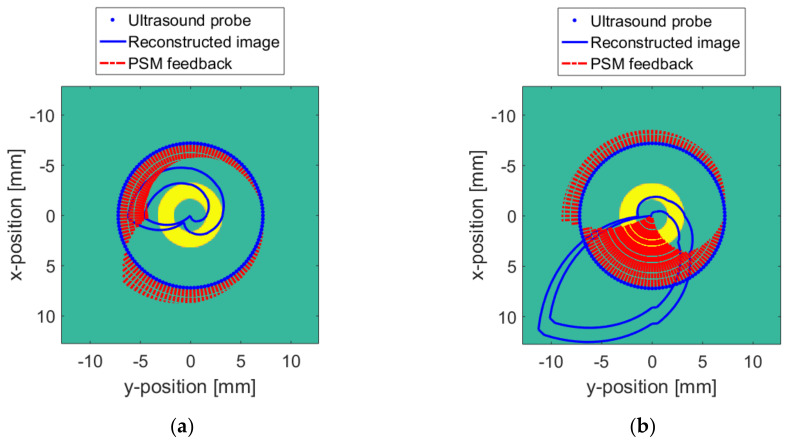
The PSM feedback when ∆v = 128: (**a**) The surface changes; (**b**) The TMC changes.

The data of the wireless probe positioning ball, attitude sensor and pressure sensor mentioned above are processed according to the range of the surface and medium. Based on these data, the PSM monitors the change in distance between the probe and the target, and the force between the probe and the surface. The changes in the distance and the force both feed back to the wireless probe for adjusting the working parameters. As shown in Figure 20, the reconstruction of the surface changes is displayed in Figure 20a, and the reconstruction of the medium changes is displayed in Figure 20b. The red dotted line in the figure indicates the feedback information of the PSM output to the wireless probe in 3D space coordinates. The length of the dashed line indicates the feedback intensity of the probe at this point. The greater the intensity, the greater the distortion of the reconstructed image at this location. In this way, the distortion of the reconstructed image can be judged in advance. Therefore, it can be corrected during the reconstruction process to obtain a more accurate reconstructed image.

**Figure 21:**
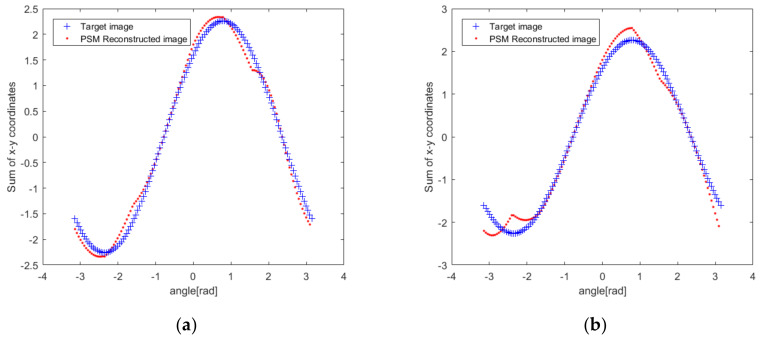
The target curve comparison: (**a**) With surface changes; (**b**) With TMC changes.

**Figure 22:**
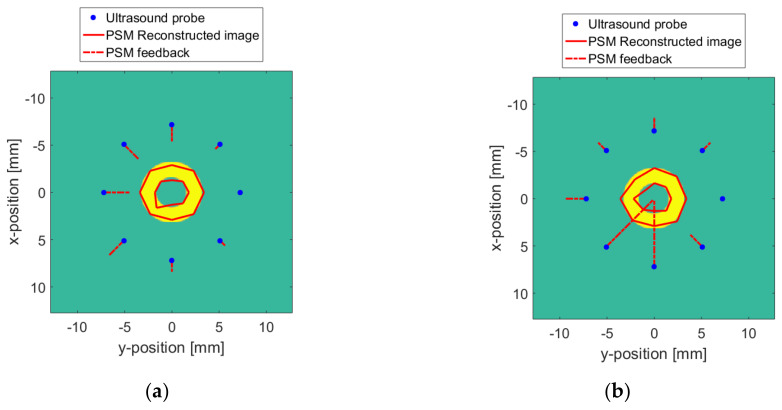
Reconstructed image with the PSM when Δv = 8: (**a**) With surface changes; (**b**) With TMC changes.

**Figure 23:**
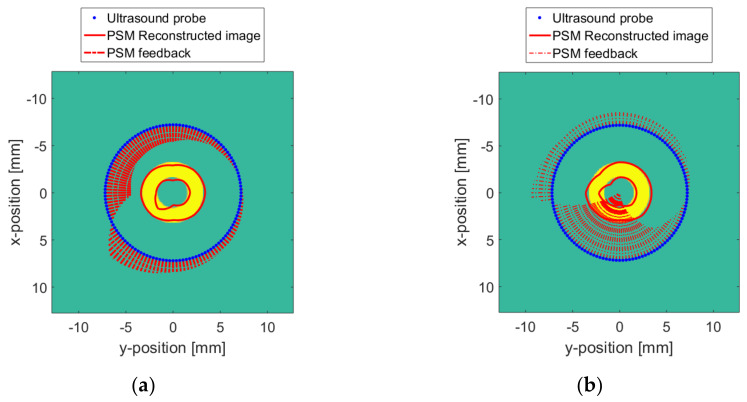
Reconstructed image with the PSM when Δv = 128: (**a**) With surface changes; (**b**) With TMC changes.

According to the PSM feedback, the reconstruction distortion due to the surface and medium changes is improved. The *X* and *Y* coordinates of each point in the ideally reconstructed image obtained in Figure 15 are considered. Additionally, the accumulated values of *X* and *Y* are taken as the vertical axis, and the radians, as the horizontal axis. The reconstructed ideal target curve in Figure 21 is obtained, as shown by the blue “+” in the figure. The reconstructed image curve corrected by the PSM is plotted with red dots. In Figure 21, a reconstructed image with a surface change is shown in Figure 21a, and a reconstructed image with a medium change is shown in Figure 21b. It can be seen that the coordinate point curve of the reconstructed image after the PSM is basically consistent with the change in the ideal case. Due to the spatial positioning error of the wireless probe and the non-linear relationship between the pressure feedback and the medium change, at some points, there is a certain deviation. But the true shape of the target can be clearly appreciated. The images reconstructed by the PSM are shown in Figure 22 and Figure 23. Figure 22 is the case where the resolution is 8. Figure 23 is the case with a resolution of 128. The red line indicates the reconstructed image. Compared with Figure 17 and Figure 19, the quality of the reconstruction of the target has been significantly improved.

### 5.3. Real Target Reconstruction Result

**Figure 24:**
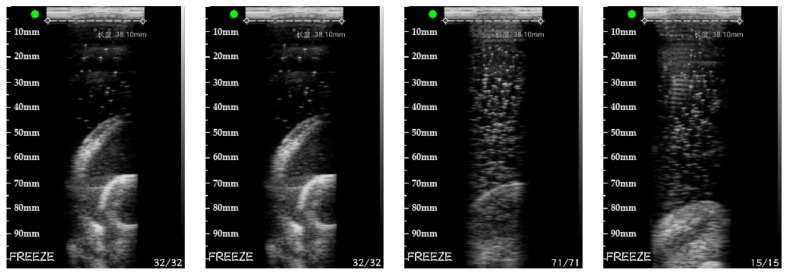

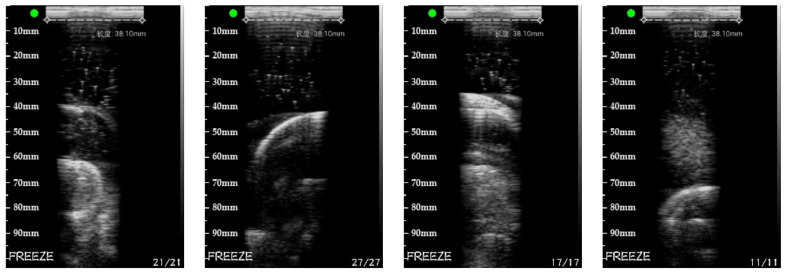
Cross-sectional images collected by the probe.

**Figure 25:**
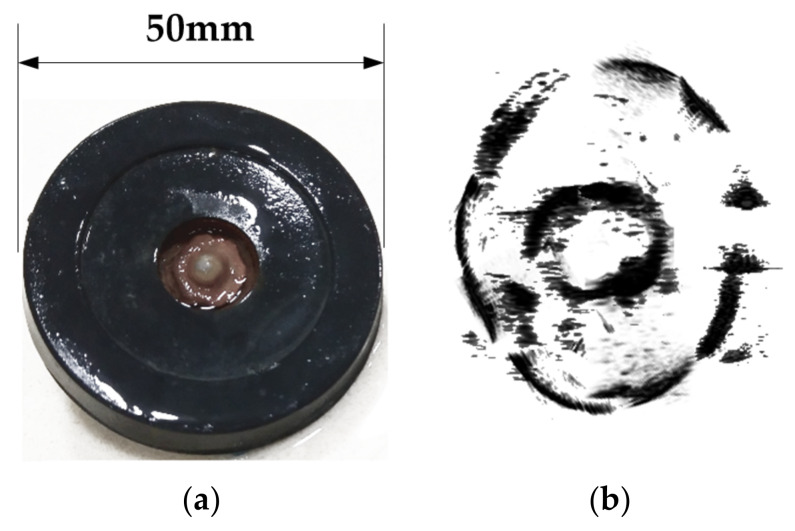
Reconstructed image of the real target with the PSM: (**a**) The real target; (**b**) The reconstructed image.

The real target experimental data are shown in Figure 24. When acquiring images, because it is a freehand probe, the distance between the probe and the target is uncertain. The medium of the container is also non-uniform. Figure 24 is a set of the cross-sectional images collected by the probe at various points in space. Figure 25 is the reconstructed image of the real target with the PSM.

## 6. Conclusions

This paper proposes a freehand 3D ultrasound reconstruction method, PSM, based on linear ultrasound transducers. Through the three steps described above, the PSM can match the current probe sector plane to a certain plane in the Sector Database. Then, the image of the probe sector is placed in a 3D space to complete the 3D ultrasound reconstruction. The PSM is validated in two experiments: one with a numerical model and the other with a real physical model. In the case of the surface extrusion and medium change, the experimental results show that the PSM effectively reduces the distortion caused by the shifted surface after pressing or the inhomogeneity of the transmission medium.

There are limitations of the study. The reconstruction results are still different from the ideal situation. This is mainly due to the errors in the spatial positioning of the probe and the non-linearity between the pressure feedback and the change of the medium. To address the problems, in the future, further study to optimize the PSM can be done in the following aspects:

1. More realistic models for the surface and the medium changes.
2. A better pressure feedback device to account for multidimensional pressure.
3. Animal experiments that may help to further validate the PSM for applications in biological sciences.

## Figures and Tables

**Table 1:** Steps of space plane inverse operation.

STEP1	According to the target position, set the probe detection depth parameter ***r***. Then, set the target point ***P_i_** (**x**, **y**, **z**)* inside the measured target. The direct distance ***D*** between ***P_i_** (**x**, **y**, **z**)* and the probe may change during the measurement procedure. The actual value of ***D*** is obtained by the spatial position of the probe and pressure compensation, which will be analyzed in detail later. The value of ***D*** is generally much larger than ***l*** which is the radius of the measured target ***M*** as shown in Figure 4.
STEP2	Select any straight line *L* that is passing through *P_i_ (x, y, z)* and parallel to the plane of the movement of the freehand probe.
STEP3	According to real-time requirements and the situation regarding data storage space, the value of the vertical resolution Δ𝑣 is determined and the number of sectors is set. A point on each sector is taken to form a set of spatial points *S_1_ (x_1_, y_1_, z_1_),…,S_n_ (x_n_, y_n_, z_n_ ) where *n = *Δ𝑣*. Using Equation (2), the space plane inverse operation can be completed to obtain the sectors *S_1_,…,S_n_*. The data for these sectors will be used to create the Sector Database (SD), which is used for the quick matching step later on.

**Table 2:** Restrictions of values.

The Angle Between the Straight Line and the Positive Direction of the Z-Axis	$\theta \in \left(0,\pi \right)$
The projection of the line on the XY plane and the positive angle of the X-axis	$\varphi \in \left(-\pi ,+\pi \right)$
The distance from the origin to the target point	$r\in \left(0,Max\left(\left|\{P_{1}\left(x_{1},y_{1},z_{1}\right),\ldots ,P_{n}\left(x_{n},y_{n},z_{n}\right)\right|\right)\right)$

**Table 3:** Steps of the improved 3D Hough transform.

STEP1	Take *B* and *C* on the current plane of the probe sector.
STEP2	Select a plane and take point *A* from the Sector Database (SD).
STEP3	The randomly generated points *A*, *B* and *C* are respectively subjected to the 3D Hough transform. If the result is a point in the parameter space, the current sector plane *P*_1_ of the probe corresponds to *S*_3_ of the database. Conversely, if the result is not a point, then repeat STEP2. If there is no corresponding plane after traversing all the sectors of the database, a re-establishment of the spatial plane model is needed and the vertical resolution Δ𝑣 is improved.

**Table 4:** Specification of wireless linear ultrasound probe.

Parameter	Value
Elements	128
Dimension	156 × 60 × 20 mm
Image frame rate	18 frames/s
Weight	220 gs ~ 250 g

**Table 5:** Initial values when the surface changes.

Δ*v* = 8, Speed of Sound = 1500 m/s Ultrasound Frequency = 1 kHz, *n* = 4 mm		
D/mm	T/us	∆t/us
1.75	1.2	−1.5
2.5	1.7	−1
3.25	2.2	−0.5
4	2.7	0
4.75	3.2	0.5
5.5	3.7	1
6.25	4.2	1.5
7	4.7	2

**Table 6:** Initial values when the surface changes.

Δ*v* = 8, Speed of Sound = 1500 m/s Ultrasound Frequency = 1 kHz, *n* = *D* = 4 mm		
TMC	T/us	∆t/us
2.77	11.2	8.5
2.04	7.7	5
1.28	4	1.3
1	2.7	0
0.85	2	−0.7
0.82	1.8	−0.9
0.81	1.7	−1
0.75	1.5	−1.2
